# A Cold-Adapted Chitinase-Producing Bacterium from Antarctica and Its Potential in Biocontrol of Plant Pathogenic Fungi

**DOI:** 10.3390/md17120695

**Published:** 2019-12-10

**Authors:** Kezhen Liu, Haitao Ding, Yong Yu, Bo Chen

**Affiliations:** 1 College of Marine Science, Shanghai Ocean University, Shanghai 201306, China; 18043425667@163.com; 2 MNR Key Laboratory for Polar Science, Polar Research Institute of China, Shanghai 200136, China; yuyong@pric.org.cn

**Keywords:** Antarctica, chitinase, cold-adapted, optimization, antifungal, *Pseudomonas*

## Abstract

To obtain chitinase-producing microorganisms with high chitinolytic activity at low temperature, samples collected from Fildes Peninsula in Antarctica were used as sources for bioprospecting of chitinolytic microorganisms. A cold-adapted strain, designated as GWSMS-1, was isolated from marine sediment and further characterized as *Pseudomonas*. To improve the chitinase production, one-factor-at-a-time and orthogonal test approaches were adopted to optimize the medium components and culture conditions. The results showed that the highest chitinolytic activity (6.36 times higher than that before optimization) was obtained with 95.41 U L^−1^ with 15 g L^−1^ of glucose, 1 g L^−1^ of peptone, 15 g L^−1^ of colloid chitin and 0.25 g L^−1^ of magnesium ions contained in the medium, cultivated under pH 7.0 and a temperature of 20 °C. To better understand the application potential of this strain, the enzymatic properties and the antifungal activity of the crude chitinase secreted by the strain were further investigated. The crude enzyme showed the maximum catalytic activity at 35 °C and pH 4.5, and it also exhibited excellent low-temperature activity, which still displayed more than 50% of its maximal activity at 0 °C. Furthermore, the crude chitinase showed significant inhibition of fungi *Verticillium dahlia* CICC 2534 and *Fusarium oxysporum* f. sp. *cucumerinum* CICC 2532, which can cause cotton wilt and cucumber blight, respectively, suggesting that strain GWSMS-1 could be a competitive candidate for biological control in agriculture, especially at low temperature.

## 1. Introduction

Chitin is a polysaccharide consisting of β-*N*-acetyl-d-glucosamine (GlcNAc) units linked by β-1,4 glycosidic bonds [1]. Chitin is a major resource for the preparation of chitin oligosaccharides, chitosan oligosaccharides and other chitin derivatives, which have tremendous applicable values in the fields of medicine, food, health care and environmental protection [2]. Generally, chitin can be decomposed through physical, chemical or biological approaches [3]. Although physical and chemical methods have been used broadly, both of them have many invincible drawbacks such as low yield, high cost, poor product uniformity and environmental pollution, while a biological method possesses the advantages of mild reaction condition, good yield, high product uniformity and environmental friendliness, especially for the enzymatic method implemented by chitinase [4].

Chitinases, which are capable of hydrolyzing chitin to release GlcNAc and *N*-acetyl chitin oligosaccharides [5], have been found in many organisms, including bacteria [6], fungi [7], plants [8], insects [9] and even humans [10]. Chitinases and chitinase-producing microorganisms have received considerable attention due to their potential applications in biological control of fungal pathogens [11] and preparation of chitin derivatives [4] in recent years. Although plenty of chitinase-producing microorganisms have been discovered and characterized, such as *Sanguibacter antarcticus* KOPRI 21702 [12], *Basidiobolus ranarum* [13], *Bacillus pumilus* U5 [14], *Chitinolyticbacter meiyuanensis* SYBC-H1 [15], *Paenibacillus* sp. D1 [16], *Serratia Marcescens* XJ-01 [17], *Streptomyces* sp. ANU 6277 [18], *Lysinibacillus fusiformis* B-CM18 [19], *Streptomyces griseorubens* C9 [20], *Streptomyces pratensis* KLSL55 [21], *Humicola grisea* ITCC 10360.16 [22], *Cohnella* sp. A01 [23], *Serratia marcescens* JPP1 [24] and *Stenotrophomonas maltophilia* [25], their chitinolytic activities are still fairly low, especially at low and intermediate temperatures, which leads to the high cost and limited large-scale application of chitinases or chitinase-producing microorganisms. As a rule of thumb, cold-adapted enzymes usually display higher activity than their mesophilic and thermophilic counterparts at the same temperature [26]. Such enzymes can be more easily found in Antarctica, a natural resource pool of cold-adapted microorganisms [27].

To obtain chitinase-producing microorganisms with high chitinolytic activity, samples collected from Fildes Peninsula on King George Island of Antarctica were used as sources for bioprospecting of chitinolytic microorganisms. The production of chitinase of the selected strain was optimized by statistical design. Besides, enzymatic properties and antifungal potential of the extracellular chitinase secreted by the strain were also investigated in this study.

## 2. Results

### 2.1. Screening, Isolation and Identification of the Chitinase-Producing Bacterium

**Figure 1:**
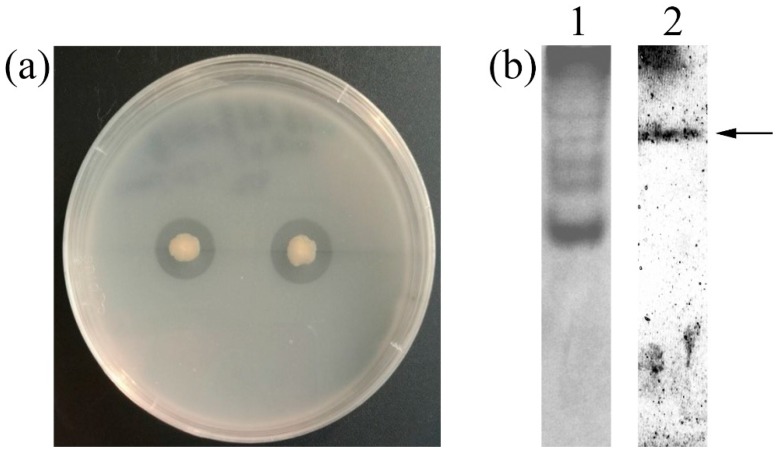
Screening and confirmation of the chitinase-producing bacterium. (**a**) Inoculation of strain GWSMS-1 on colloidal chitin plate. (**b**) Native-PAGE of concentrated crude chitinase secreted by strain GWSMS-1. In lane 1, the gel was stained by Coomassie Brilliant Blue R-250. In lane 2, the gel was stained by Calcofluor White M2R. The proposed chitinase was indicated by an arrow.

Strain GWSMS-1, isolating from marine sediment, produced a clear transparent zone on the colloidal chitin plate (Figure 1a), indicating that it is capable of secreting chitinase to hydrolyze the colloidal chitin around itself. The native-PAGE was conducted to further verify the chitinase activity of the secreted enzyme. As shown in Lane 2 of Figure 1b, a clear band was observed on the gel, implying the presence of chitinase in the crude enzyme secreted by strain GWSMS-1. 

**Figure 2:**
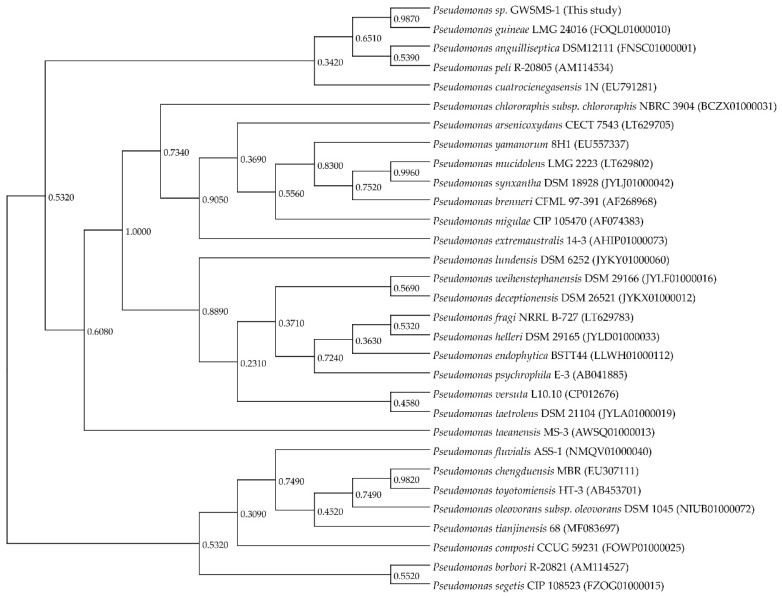
Phylogenetic analysis based on 16S rDNA sequences of *Pseudomonas* sp. GWSMS-1 and its phylogenetically related species. The GenBank accession number is provided following the species name.

Strain GWSMS-1 was classified into genus *Pseudomonas* by molecular identification using 16S-rDNA sequencing. To understand the evolutionary relationship between *Pseudomonas* sp. GWSMS-1 and its phylogenetically related species, a 16S rDNA-based phylogenetic analysis was conducted using a total of thirty-one 16S rDNA gene sequences retrieved from EzBioCloud web server [28]. The 16S rDNA of strain GWSMS-1 showed the highest similarity (99.79%) with *Pseudomonas guineae* LMG 24016 [29], a psychrotolerant bacterium also isolated from Antarctica (Figure 2). Since these two strains occupied a distinct position in genus *Pseudomonas*, it is suggested that they might experience a similar evolutionary journey to adapt to the extreme environment of Antarctica.

### 2.2. One-Factor-at-a-Time Optimization

**Figure 3:**
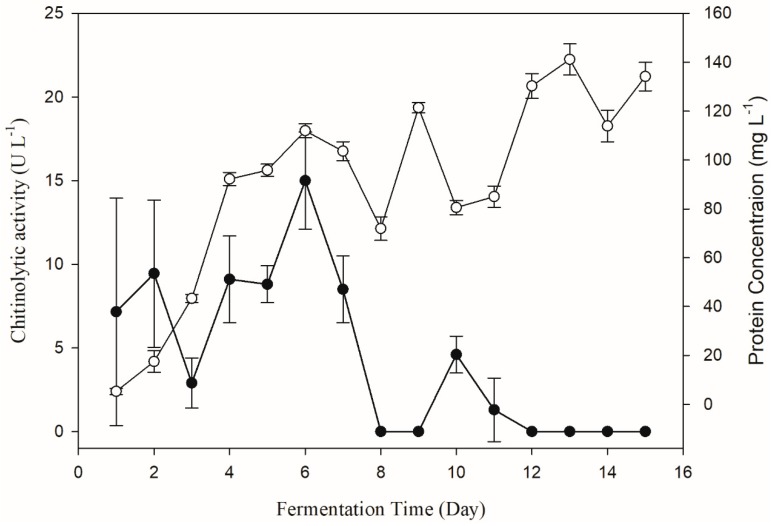
Changes in the chitinolytic activity of *Pseudomonas* sp. GWSMS-1 during the fermentation process. Chitinolytic activity and protein concentration are represented as solid and empty circles, respectively.

The change of chitinolytic activity of *Pseudomonas* sp. GWSMS-1 during the fermentation process was monitored to determine the fermentation time for chitinase production with the highest activity. As shown in Figure 3, the chitinolytic activity could be detected in the fermentation broth after 24 h of cultivation, and it achieved its maximum on the sixth day. It is worth mentioning that the chitinolytic activity (solid circle) increased with the increase in protein concentration (empty circle) in the first six days, but decreased sharply with increased consumption of chitin in later days. It is proposed that the chitinase of strain GWSMS-1 is an inducible enzyme, which could only be produced in the presence of chitin with high enough concentration. Therefore, fermentation broth cultivated for 6 days was used for measuring the chitinolytic activity in further study.

**Figure 4:**
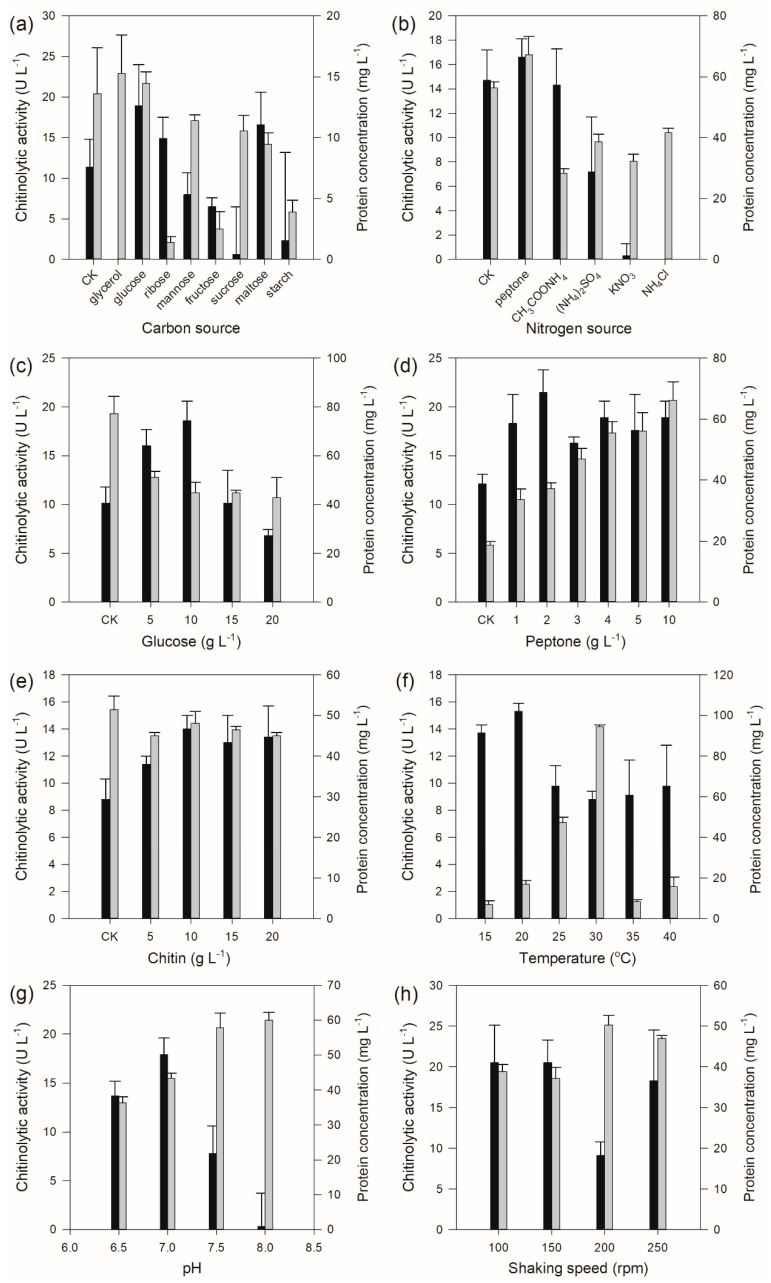
Chitinase production of *Pseudomonas* sp. GWSMS-1 optimized by the one-factor-at-a-time method. Effects of (**a**) carbon source, (**b**) nitrogen source, (**c**) glucose concentration, (**d**) peptone concentration, (**e**) chitin concentration, (**f**) temperature, (**g**) pH and (**h**) shaking speed on the chitinase production. Chitinolytic activity and protein concentration are represented as black and grey bars, respectively.

The results of carbon source selection showed that the carbon source exerted a significant influence on the chitinolytic activity, which was undetectable when glycerol was used as carbon source, while the strain produced the highest amount of chitinolytic activity when glucose was used as carbon source (Figure 4a) with a concentration of 10 g L^−1^ (Figure 4c). Nitrogen source test results displayed that the organic nitrogen sources had a better effect on chitinolytic activity than those of inorganic nitrogen sources (Figure 4b). The highest chitinolytic activity was determined from the fermentation broth when peptone was employed as the nitrogen source with a concentration of 2 g L^−1^ (Figure 4d). In addition, different chitin concentrations also affected the production of chitinase by *Pseudomonas* sp. GWSMS-1, and the highest apparent yield of the enzyme was observed when the chitin concentration was 10 g L^−1^ (Figure 4e). The fermentation condition optimization showed that the optimum temperature, pH and shaking speed for the production of chitinolytic enzymes were determined as 20 °C (Figure 4f), 7.0 (Figure 4g) and 100–150 rpm (Figure 4h), respectively. 

### 2.3. Orthogonal Design

With the aim of obtaining more chitinase secreted by strain GWSMS-1, the medium components were further optimized by orthogonal design. The results showed that the apparent highest chitinolytic activity of 72.16 U L^−1^ was obtained with the seventh combination (Table 1). Further analysis of the data implied that the desired highest activity would be achieved when the concentrations of glucose, peptone, colloid chitin and magnesium ions are 15 g L^−1^, 1 g L^−1^, 15 g L^−1^ and 1 mM, respectively. Subsequently, an additional experiment was performed to verify this combination, which was not included in the orthogonal test. Finally, the chitinolytic activity was determined as 95.41 U L^−1^ with the above combination, which was higher than the apparent highest activity (72.16 U L^−1^) observed in the orthogonal test. In variance analysis, F_0.01_ = 6.23 is used as a reference value, and F > 6.23 means a significant effect of the factor. According to the variance analysis of the orthogonal test showed in Table 2, all these four factors involved in the optimization showed significant effects on the yield of chitinase at the *p* = 0.01 level, and peptone and chitin were the most significant factors.

Therefore, the final medium for chitinase production of *Pseudomonas* sp. GWSMS-1 was determined as follows (L^−1^): glucose 15 g, peptone 1 g, colloidal chitin 15 g, MgSO_4_·7H_2_O 0.25 g, KH_2_PO_4_ 0.3 g, K_2_HPO_4_·3H_2_O 1 g. 

### 2.4. Temperature and pH-Dependent Enzymatic Properties of Chitinase

**Figure 5:**
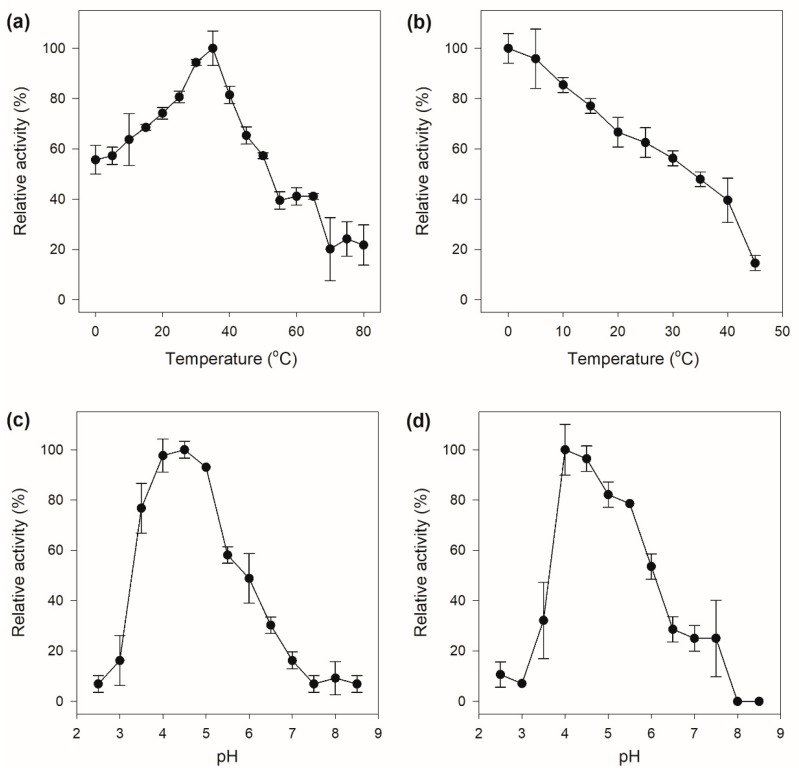
Enzymatic properties of the crude chitinase. (**a**) Optimal temperature; (**b**) temperature stability; (**c**) optimal pH; (**d**) pH stability.

The crude chitinase showed chitinolytic activity in a wide temperature range with the maximum catalytic activity at 35 °C. Furthermore, the enzyme also exhibited excellent low-temperature activity, which still displayed more than 50% of its maximal activity at 0 °C (Figure 5a). A generally accepted hypothesis is that high low-temperature activity of cold-adapted enzymes evolved to facilitate binding and conversion of the substrate at low temperatures, which is consistently accompanied by weak thermal stability on account of the intrinsic structural flexibility of the enzymes, which is supposed to be a result of evolutionary pressure [30]. The crude enzyme was only stable at low temperature and was rapidly inactivated with increasing temperature (Figure 5b). The crude chitinase had a high chitinolytic activity between pH 4.0–5.0, with an optimum catalytic activity at pH 4.5 (Figure 5c), indicating that the chitinase might be an acidic enzyme. The pH stability of the crude chitinase exhibited a similar pattern to that of the activity response to pH, which was stable in the pH range of 4.0 to 5.0 and rapidly deactivated under other pH values (Figure 5d). 

### 2.5. Antifungal Activity

**Figure 6:**
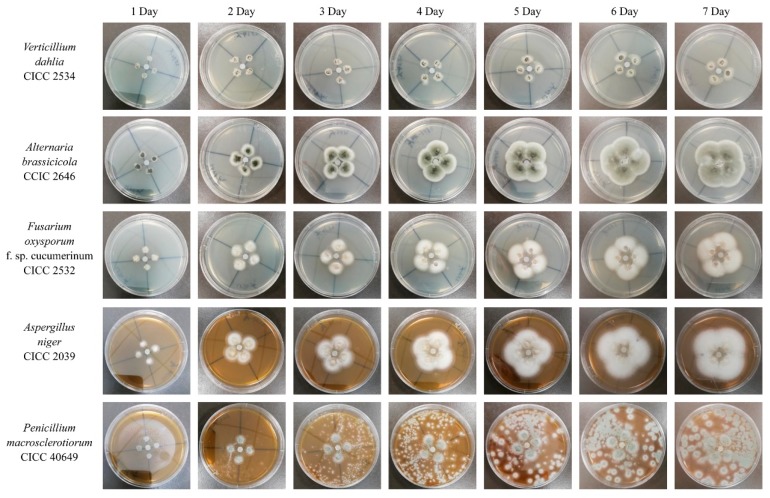
Antifungal activity of the chitinase secreted by *Pseudomonas* sp. GWSMS-1.

As a key component of fungal cell wall, chitin is essential for fungal pathogens to maintain their cell structure integrity. Considering that chitinase is capable of degrading chitin to decompose the fungal cell wall, it is indispensable to evaluate the antifungal potential of strain GWSMS-1. However, since strain GWSMS-1 is not one of GRAS (generally regarded as safe) strains, it cannot be applied to the medical field without any safety tests, whereas the requirement is less stringent for agricultural application. Therefore, five common phytopathogenic fungi were selected to evaluate the potential application in biocontrol of strain GWSMS-1. As shown in Figure 6, the crude enzymes significantly inhibited the phytopathogenic fungi *Verticillium dahlia* CICC 2534 and *Fusarium oxysporum* f. sp. *cucumerinum* CICC 2532 and slightly inhibited *Aspergillus niger* CICC 2039 and *Penicillium macrosclerotiorum* CICC 40649, even after incubation for 7 days, while not showing any inhibition toward *Alternaria brassicicola* CICC 2646 during the entire incubation. 

## 3. Discussion

In this study, a chitinase-producing strain GWSMS-1 was isolated from marine sediment near the China’s Great Wall Station in Antarctica and characterized as a member of genus *Pseudomonas*. Statistical optimization of the chitinase production, the enzymatic properties and the antifungal activity of the chitinase was conducted for better evaluating the application potential of this strain.

Enzyme production is one of the most important limitations for the large-scale application of enzymes, which significantly affects the usage cost. Generally, the yield of an enzyme is optimized from two aspects: medium indigents and culture conditions. In this study, the optimum culture conditions, including temperature, pH and shaking speed, were determined as 20 °C, 7.0 and 150 rpm, respectively, which shared similar conditions except for temperature with other strains reported previously (Table 3). It is obvious that the optimum temperatures for the secretion of chitinase by different strains are associated with their optimum growth temperature. Dissolved oxygen level, represented by shaking speed, has little effect on chitinase production among different strains. Another noticeable difference in culture conditions among different chitinase-producing strains is the fermentation time, which ranged from 1 to 8 days (Table 3). Comparing with mesophilic microorganisms, *Pseudomonas* sp. GWSMS-1 has a relatively long fermentation time to achieve its maximum yield of chitinase as a cold-adapted microorganism. However, some mesophilic and thermophilic strains, such as *Streptomyces griseorubens* C9 [20], *Bacillus pumilus* U5 [14] and *Humicola grisea* ITCC 10360.16 [22], also showed similar fermentation periods to GWSMS-1, which might be due to their intrinsic regulation of metabolism. In the further study, strain GWSMS-1 could be genetically modified by metabolic engineering to reduce the fermentation time in order to make the fermentation process economical.

Since only a few strains, such as *Thermococcus chitonophagus* [31], *Microbispora* sp. V2 [32] and *Metarrhizium anisopliae* [33], can utilize chitin as the sole carbon source, most of the chitinolytic microorganisms cannot produce chitinolytic enzymes with chitin as the sole carbon source. Therefore, it is necessary to add additional carbon sources that are feasible to utilize by these strains through co-metabolism (Table 3). The additional carbon source mainly provides energy for cell growth and proliferation as the primary matrix, while chitin is decomposed and utilized as the secondary matrix. In general, glucose is the best carbon source for enzyme production, and strain GWSMS-1 is no exception. However, other small molecules such as lactose and glycerol showed a better effect on chitinase secretion than glucose in some cases (Table 3). Unlike the variety of carbon source preferences of different strains, almost all the studies showed that the organic nitrogen source is better for enzyme production than the inorganic nitrogen source (Table 3), which inhibited the synthesis of chitinase during fermentation; organic nitrogen was better for strain GWSMS-1 as well. 

The potential application in biocontrol of fungi of the cold-active chitinase secreted by strain GWSMS-1 was evaluated using five common plant pathogens. The crude chitinase showed significant inhibition on fungi *Verticillium dahlia* CICC 2534 and *Fusarium oxysporum* f. sp. *cucumerinum* CICC 2532 which can cause cotton wilt and cucumber blight, respectively, suggesting that strain GWSMS-1 would be a competitive candidate for the biological control in agriculture.

## 4. Materials and Methods

### 4.1. Chemicals, Agents and Media

Chitin, potassium ferricyanide and *N*-acetyl-d-glucosamine (NAG) were available from Sigma-Aldrich (St. Louis, MO, USA). All other chemicals of analytical grade were purchased from Sangon Biotech (Shanghai, China). 

Colloidal chitin was prepared according to Souza et al. [34] as follows: five grams of chitin powder was added to 60 mL of concentrated HCl slowly and incubated overnight, with vigorous stirring at room temperature. The mixture was added to 200 mL precooling ethanol and incubated at room temperature overnight with vigorous stirring. The precipitate was harvested by centrifugation at 5000 *g* for 20 min at 4 °C. The colloidal chitin was washed with sterile distilled water to neutral and stored in the dark at 4 °C. 

Potassium ferrocyanide solution was prepared by dissolving 0.5 g potassium ferrocyanide in 1 liter of 0.5 M Na_2_CO_3_ buffer and stored in a dark environment. 

The initial liquid medium consisted of peptone (2 g L^−1^), glucose (1 g L^−1^), colloid chitin (5 g L^−1^), FeSO_4_·7H_2_O (0.01 g L^−1^), MgSO_4_·7H_2_O (0.5 g L^−1^), KH_2_PO_4_ (0.3 g L^−1^) and K_2_HPO_4_·3H_2_O (0.917 g L^−1^), while the solid medium contained agar at a concentration of 1.5%.

### 4.2. Screening and Characterization of Chitinase-Producing Microorganisms

Samples of seal and penguin feces, soil and marine sediment, collected from Fildes Peninsula (60°20’S–60°56’S, 44°05’W–46°25’W) in Antarctica, were used as sources for bioprospecting of chitinase-producing microorganisms. Strains which formed transparent zones on the colloidal chitin plate at 15 °C were selected for further study. The isolates were characterized by 16S rDNA sequencing. The 16S rDNA gene was amplified using genomic DNA as templates, with universal primer pairs 27F (5’-AGAGTTTGATCMTGGCTCAG-3’ (27F) and 1492R (5’-TACGGYTACCTTGTTACGACTT-3’). 

The PCR products were ligated with the pMD19-T vector and transformed into *E. coli* DH5α competent cells for sequencing. The nucleotide sequence of the 16S rDNA gene was subject to BLAST server (http://www.ncbi.nlm.nih.gov/BLAST) to find homologous sequences. Multiple sequence alignment was conducted using the software Clustal X 2.0 [35]. The phylogenetic tree was constructed using the neighbor-joining method [36] in MEGA 6.0 [37], with a bootstrap test of 1000 replicates.

### 4.3. Preparation of Crude Chitinase

The fermentation broth was centrifuged at 8000× *g* for 10 min, and the supernatant was concentrated by using a 10 kDa ultrafiltration centrifuge tube. The eluate was then filtered through a 0.22 μm filter and stored at −20 °C for further experiments. 

### 4.4. Native-PAGE and Active Staining of Chitinase

To obtain enough crude chitinase for native-PAGE, the crude chitinase was further concentrated as follows: the crude enzyme was mixed with appropriate amount of colloidal chitin and incubated at 4 °C for 2 h; the mixture was washed twice with 50 mM of Tris-HCl (pH 8.0), then the concentrated chitinase was eluted by using 50 mM of acetate buffer (pH 4.0) and dialyzed by using 50 mM of Tris-HCl (pH 8.0). The native-PAGE was performed using 4% stacking gel and 12% separating gel with 0.5% colloidal chitin added. The gel was stained by Coomassie Brilliant Blue R-250 and Calcofluor White M2R [38] to verify the chitinase activity of the crude enzyme. 

### 4.5. Chitinase Activity Assay

Chitinase activity was determined by measuring the amount of NAG generated from colloidal chitin using a potassium ferrocyanide solution, according to Taiji et al. [39]. An appropriate amount of crude extracellular chitinase secreted by strain GWSMS-1 was mixed with colloidal chitin (1%, *m*/*v*) suspended in 50 mM phosphate buffer at pH 6.0. The mixture of enzyme and substrate was incubated at 30 °C for 2 h, then treated at 100 °C for 5 min to inactivate the enzyme. Subsequently, the reaction solution was centrifugated at 10,000× *g* for 5 min to remove the precipitate, and 0.05 mL of supernatant was mixed with 1.45 mL potassium ferrocyanide solution. The absorbance of the mixture at 420 nm was measured after treating at 100 °C for 15 min and cooling to room temperature. The NAG concentration was calculated based on the standard curve obtained under the same condition. One unit of chitinase activity was defined as the amount of enzyme required to produce 1 μmol of NAG per minute at 30 °C in a phosphate buffer at pH 6.0.

### 4.6. One-Factor-at-a-Time Optimization 

To obtain maximum extracellular chitinase secreted by strain GWSMS-1, the one-factor-at-a-time method was adopted to optimize the medium composition including carbon and nitrogen sources, carbon, nitrogen and chitin concentration, as well as the culture conditions including fermentation time, temperature, pH and shaking speed (Table 4). All experiments were performed in triplicate.

### 4.7. Orthogonal Design

Based on the results of the single factor test, the medium composition was further optimized by orthogonal design. The orthogonal test employed a four-factor and three-level orthogonal table L_9_ (3^4^) to optimize concentrations of glucose, peptone, chitin and magnesium ions (Table 5). All experiments were performed with three replicates.

### 4.8. Temperature and pH-Dependent Enzymatic Properties of Crude Chitinase 

Generally, the activity and stability of enzymes can be determined by pH denaturation and thermal denaturation [40]. The optimal temperature of the crude chitinase was determined by assaying the activity at different temperatures ranging from 0 to 80 °C with 5 °C intervals at pH 6.0. The thermal stability was determined by measuring the residual activity after treating the crude enzyme at different temperatures from 0 to 45 °C with 5 °C intervals at pH 6.0 for 30 min. The optimum pH for the crude chitinase was determined by measuring the activity in acetate buffer (pH 2.5–3.5), citric acid buffer (pH 4.0–5.5), phosphate buffer (pH 6.0–7.5), Tris-HCl buffer (pH 8.0–9.0) and glycine–NaOH buffer (pH 9.5–11.0) at 30 °C. The pH stability was assayed by measuring the residual activity after incubating the crude enzyme in the buffers mentioned above at 30 °C for 30 min.

### 4.9. Antifungal Activity Assay 

The antifungal activity of the extracellular chitinase secreted by strain GWSMS-1 was investigated by hyphal extension inhibition. Hyphal extension inhibition assay was estimated by the paper disk method. Filter papers with a 6 mm diameter were immersed in the concentrated crude enzyme solution for 5 min. A piece of soaked filter paper was placed at the center of the petri dishes containing potato dextrose agar (PDA). The mycelium of the test fungi was inoculated around the filter paper and incubated at 20 °C for 7 days for the mycelia to grow. The heat-inactivated crude enzyme was used as a control. Fungi used in this study were purchased from China Center of Industrial Culture Collection (CICC) (Beijing China), including *Verticillium dahlia* CICC 2534, *Alternaria brassicicola* CICC 2646, *Fusarium oxysporum* f. sp. *cucumerinum* CICC 2532, *Aspergillus niger* CICC 2039 and *Penicillium macrosclerotiorum* CICC 40649.

## 5. Conclusions

In this study, a cold-adapted chitinase-producing strain GWSMS-1 was isolated from marine sediment and characterized as *Pseudomonas*. Strategy coupling of the one-factor-at-a-time and the orthogonal test was employed to optimize the chitinase production of the strain. The optimized production was about 6.36 times higher than that before optimization. Based on the biochemical characterization, the crude chitinase was determined as a typical cold-active enzyme, which exhibited excellent low-temperature activity at 0 °C. In addition, it also showed significant inhibition of two plant pathogens, suggesting that strain GWSMS-1 would be a competitive candidate for the biological control in agriculture, especially in high latitudes.

## Figures and Tables

**Table 1:** Orthogonal design and the responding chitinolytic activity.

No.	(A) Glucose (g L^−1^)	(B) Peptone (g L^−1^)	(C) Chitin (g L^−1^)	(D) Mg^2+^ (mM)	Chitinolytic Activity (U L^−1^)
1	5	1	5	1	52.25 ± 3.73
2	5	2	10	5	16.17 ± 2.16
3	5	3	15	10	22.64 ± 6.26
4	10	1	10	10	18.66 ± 3.73
5	10	2	15	1	51.01 ± 2.15
6	10	3	5	5	7.46 ± 6.47
7	15	1	15	5	72.16 ± 7.77
8	15	2	5	10	37.32 ± 3.73
9	15	3	10	1	6.22 ± 2.16
*K1*	91.07	143.08	97.04	109.49	
*K2*	77.14	104.51	41.06	95.80	
*K3*	115.71	36.33	145.81	78.63	
*k1*	30.36	47.69	32.35	36.50	
*k2*	25.71	34.84	13.69	31.93	
*k3*	38.57	12.11	48.60	26.21	
Range	12.86	35.58	34.91	10.29	
Factor order	B > C > A > D				
Optimization combination	A3	B1	C3	D1	

**Table 2:** Analysis of Variance (ANOVA).

Source	Sum of Square	Degrees of Freedom	Mean Square	F-value	*p*-value
Glucose	762.85	2	381.42	17.42	<0.01
Peptone	5843.69	2	2921.84	133.43	<0.01
Chitin	5495.72	2	2747.86	125.49	<0.01
Mg^2+^	478.03	2	239.02	10.92	<0.01
Error	394.16	18	21.90		
Total	39843.59	27			

**Table 3:** Summary of the optimized liquid fermentation conditions of chitinase-producing microorganisms.

Strains	Source	Method	Component ^a^ (g L^−1^)	Condition	Yield (Final/ Initial)
*Pseudomonas* sp. GWSMS-1 (this study)	Sediments, Antarctic	OFAT OD	Colloidal chitin: 15.0 Glucose: 15.0 Peptone: 1.0 MgSO_4_·7H_2_O: 0.25 KH_2_PO_4_: 0.3 K_2_HPO_4_·3H_2_O: 1.0	Temperature: 20 °C pH: 7.0 Rotary speed: 150 rpm Time: 6 days	6.36
*Sanguibacter* *antarcticus* KOPRI 21702 [12]	Sea sand, Antarctic	OFAT PBD RSM	Chitin: 2.0 Glycerol: 10.0 Peptone: 5.0 Yeast extract: 1.0 Fe(C_6_H_5_O_7_): 0.01 NaCl: 23.0 MgCl_2_: 2.5 Na_2_SO_4_: 3.24 CaCl_2_: 1.8 NaHCO_3_: 0.16	Temperature: 25 °C pH: 6.5 DO: 30% Time: 3 days	7.5
*Basidiobolus ranarum* [13]	Frog excrement	RSM	Colloidal chitin: 15 Lactose: 1.25 Malt extract: 0.25 Peptone: 0.75	Temperature: 25 °C pH: 9.0 Rotary speed: 200 rpm Time: 5 days	7.71
*Bacillus* *pumilus* U5 [14]	Soil, Iran	PBD RSM	Chitin: 4.760 Yeast extract: 0.439 MgSO_4_⋅7H_2_O: 0.0055 FeSO_4_⋅7H_2_O: 0.019	Temperature: 30 °C pH: 6.5 Rotary speed: 150 rpm Time: 8 days	1.20
*Chitinolyticbacter meiyuanensis* SYBC-H1 [15]	Soil, China	PBD RSM	Chitin: 3.8 Inulin: 3.55 Urea: 3.1 (NH_4_)_2_SO_4_: 0.64 MgSO_4_·7H_2_O: 0.5 FeSO_4_·7H_2_O: 0.02 KH_2_PO_4_: 0.7 K_2_HPO_4_: 0.3	Temperature: 30 °C pH: 7.0 Rotary speed: 200 rpmTime: 4 days	15.5
*Paenibacillus* sp. D1 [16]	Effluent, India	PBD RSM	Chitin: 3.75 Yeast extract: 0.6 5Urea: 0.33 MgSO_4_: 0.30 K_2_HPO_4_: 1.17	Temperature: 30 °C pH: 7.2 Rotary speed: 180 rpm Time: 3 days	2.56
*Serratia* *Marcescens* XJ-01 [17]	Fishing field, China	OFA TOD	Colloidal chitin: 7.5 (NH_4_)_2_SO_4_: 5 MgSO_4_⋅7H_2_O: 0.5 KH_2_PO_4_: 2.4 K_2_HPO_4_·3H_2_O: 0.6	Temperature: 32 °C pH: 8.0 Rotary speed: 180 rpm Time: 32 h	N.M. ^c^
*Streptomyces* sp. ANU 6277 [18]	Soil, India	OFAT	Colloidal chitin: 10.0 Starch: 2.0 Yeast extract: 4.0 KH_2_PO_4_: 2 MgSO_4_⋅7H_2_O: 1 FeSO_4_·7H_2_O: 0.1	Temperature: 35 °C pH: 6.0 Time: 2.5 days	N.M.
*Lysinibacillus fusiformis* B-CM18 [19]	Chickpea rhizosphere	OFAT RSM	Colloidal chitin: 5.50 Starch: 0.55 Yeast extract: 0.55 NaCl: 4.5 NH_4_Cl: 1.0 CaCl_2_: 0.1 MgSO_4_: 0.12 KH_2_PO_4_: 3.0 Na_2_HPO_4_: 6.0	Temperature: 32.5 °C pH: 7.0 Rotary speed: 150 rpm Time: 2–5 days	56.1
*Streptomyces* *griseorubens* C9 [20]	Soil, Algeria	PBD RSM	Colloidal chitin: 20.0 Yeast extract: 0.25 Data syrup: 4.7 K_2_HPO_4_/KH_2_PO_4_: 1.81	Temperature: 40 °C pH: natural Rotary speed: 150 rpm Time: 7 days	26.38
S*treptomyces pratensis* KLSL55 [21]	Soil, India	OFAT	Colloidal chitin: 15 Fructose: 12.5 KNO_3_: 5 Mn^2+^: 0.5	Temperature: 40 °C pH: 8.0 Rotary speed: 160 rpm Time: 2 days	14.3
*Humicola* *grisea* ITCC 10360.16 [22]	Desert soil, India	PBD RSM	Chitin: 7.49 Colloidal chitin: 4.91 Yeast extract: 5.5 KCl: 0.19 NH_4_Cl: 1.0 MgSO_4_⋅7H_2_O: 0.2 KH_2_PO_4_: 0.68 K_2_HPO_4_: 0.87	Temperature: 45 °C pH: 6.5 Rotary speed: 150 rpm Time: 8 days	1.43
*Cohnella* sp. A01 [23]	Wastewater, Iran	OFAT OD	Colloidal Chitin: 15 NH_4_NO_3_: 5 KH_2_PO_4_: 0.7 NaCl: 1.7	Temperature: 60 °C pH: 6.5 Rotary speed: 180 rpm Time: 3 days	N.M.
*Serratia* *marcescens* JPP1 [24]	Peanut hulls, China	PBD RSM	Colloidal chitin: 12.7 Glucose: 7.34 Peptone: 5.0 (NH_4_)_2_SO_4_: 1.32 MgSO_4_⋅7H_2_O: 0.5 K_2_HPO_4_: 0.7	N.M.	2.1
*Stenotrophomonas maltophilia* [25]	Soil, India	PBD RSM	Colloidal chitin: 4.94 Maltose: 5.56 Yeast extract: 0.62 KH_2_PO_4_: 1.33 MgSO_4_⋅7H_2_O: 0.65	N.M.	N.M.

OFAT, one-factor-at-a-time; OD, orthogonal design; PBD, Plackeet–Burmann Design; RSM, response surface methodology; N.M., not mentioned in the corresponding study. ^a^ The trace elements added into the medium were omitted.

**Table 4:** Factors and variables of one-factor-at-a-time optimization.

Factors	Variables
Time (days)	1, 2, 3, 4, 5, 6, 7, 8, 9, 10, 11, 12, 13, 14, 15
Carbon source	glycerol, glucose, ribose, mannose, fructose, sucrose, maltose, starch
Nitrogen source	peptone, CH_3_COONH_4_, (NH_4_)_2_SO_4_, KNO_3_, NH_4_Cl
Glucose (g L^−1^)	5, 10, 15, 20
Peptone (g L^−1^)	1, 2, 3, 4, 5, 10
Chitin (g L^−1^)	5, 10, 15, 20
Temperature (°C)	15, 20, 25, 30, 35, 40
pH	5.0, 6.0, 6.5, 7.0, 7.5, 8.0, 9.0
Shaking speed (rpm)	100, 150, 200, 250

**Table 5:** Levels of orthogonal design.

Levels	Glucose (g L^−1^)	Peptone (g L^−1^)	Chitin (g L^−1^)	Mg^2+^ (mM)
1	5	1	5	1
2	10	2	10	5
3	15	3	15	10
